# RNA-sequencing of AVPV and ARH reveals vastly different temporal and transcriptomic responses to estradiol in the female rat hypothalamus

**DOI:** 10.1371/journal.pone.0256148

**Published:** 2021-08-18

**Authors:** Margaret A. Mohr, Angela M. Wong, Gauthaman Sukumar, Clifton L. Dalgard, Weizhe Hong, T. John Wu, Ye Emily Wu, Paul E. Micevych

**Affiliations:** 1 Dept of Neurobiology, and the Laboratory of Neuroendocrinology of the Brain Research Institute, UCLA DGSOM, Los Angeles, California, United States of America; 2 Dept of Gynecological Surgery and Obstetrics, Uniformed Services University of the Health Sciences, Bethesda, Maryland, United States of America; Duke University School of Medicine, UNITED STATES

## Abstract

In females, estrogens have two main modes of action relating to gonadotropin secretion: positive feedback and negative feedback. Estrogen positive and negative feedback are controlled by different regions of the hypothalamus: the preoptic area/anterior portion (mainly the anteroventral periventricular nucleus, AVPV) of the hypothalamus is associated with estrogen positive feedback while the mediobasal hypothalamus (mainly the arcuate nucleus of the hypothalamus, ARH), is associated with estrogen negative feedback. In this study, we examined the temporal pattern of gene transcription in these two regions following estrogen treatment. Adult, ovariectomized, Long Evans rats received doses of estradiol benzoate (EB) or oil every 4 days for 3 cycles. On the last EB priming cycle, hypothalamic tissues were dissected into the AVPV+ and ARH+ at 0 hrs (baseline/oil control), 6 hrs, or 24 hrs after EB treatment. RNA was extracted and sequenced using bulk RNA sequencing. Differential gene analysis, gene ontology, and weighted correlation network analysis (WGCNA) was performed. Overall, we found that the AVPV+ and ARH+ respond differently to estradiol stimulation. In both regions, estradiol treatment resulted in more gene up-regulation than down-regulation. *S100g* was very strongly up-regulated by estradiol in both regions at 6 and 24 hrs after EB treatment. In the AVPV+ the highest number of differentially expressed genes occurred 24 hrs after EB. In the ARH+, the highest number of genes differentially expressed by EB occurred between 6 and 24 hrs after EB, while in the AVPV+, the fewest genes changed their expression between these time points, demonstrating a temporal difference in the way that EB regulates transcription these two areas. Several genes strongly implicated in gonadotropin release were differentially affected by estradiol including *Esr1*, encoding estrogen receptor-α and *Kiss1*, encoding kisspeptin. As an internal validation, *Kiss1* was up-regulated in the AVPV+ and down-regulated in the ARH+. Gene network analysis revealed the vastly different clustering of genes modulated by estradiol in the AVPV+ compared with the ARH+. These results indicate that gene expression in these two hypothalamic regions have specific responses to estradiol in timing and direction.

## Introduction

In females, estrogen signaling in the hypothalamus elicits two different feedback loops that regulate reproduction. Throughout most of the estrous cycle, low amounts of circulating estradiol exert negative feedback action on gonadotropin releasing hormone (GnRH), halting the release of gonadotrophs (luteinizing hormone, LH, and follicle stimulating hormone, FSH). However, on proestrus, rising levels of estradiol initiate positive feedback signaling, causing an increased frequency and amplitude of GnRH release eliciting a surge of LH from the pituitary gland, inducing ovulation. The primary mediator of both estrogen negative and positive feedback appears to be the kisspeptin neurons, Kisspeptin neurons in the arcuate nucleus of the hypothalamus (ARH) mediate estrogen negative feedback and the pulsatile release of GnRH [1–4], while kisspeptin neurons in the anteroventral periventricular nucleus (AVPV) mediate positive feedback [5–7]. Sex differences in these kisspeptin populations correspond to functional sex differences in estrogen feedback. Mirroring estrogen negative feedback that occurs in males and females. While there is no sex difference in number of ARH kisspeptin neurons [8], in the AVPV, females have a larger population of kisspeptin neurons, which control positive feedback, which is only present females [9].

The switch between positive and negative feedback signaling involves a balance of estradiol and progesterone signaling, with coordination of the master circadian clock, regulated by the suprachiasmatic nucleus (SCN; reviewed in [10–12]. While there is rapid regulation of the GnRH neurons involving GABA, glutamate signaling, and kisspeptin, both negative and positive feedback require estrogenic control of transcription. Because GnRH neurons do not express the reproductively critical ERα, or nuclear progesterone receptor (PGR) [13–15], steroid information to GnRH neurons must be transduced by other neurons and glial cells that express the appropriate receptors. Among the various estradiol-responsive inputs to GnRH cells, perhaps the most critical is kisspeptin.

ARH kisspeptin neurons express neurokinin B (NKB) and dynorphin and are designated KNDy neurons. These neurons appear to be the pulse generator for GnRH release and the site of negative feedback [16–18]. Estradiol inhibits ARH kisspeptin expression and removal of estradiol up-regulates kisspeptin levels [8]. Lesioning the KNDy neurons with saporin [1] blocks negative feedback [2, 19]. In contrast with the ARH kisspeptin population, kisspeptin expression is up-regulated by estradiol in the AVPV [8].

Gene expression studies have not been focused on the AVPV region, nor have they been conducted in animals treated with a hormonal replacement paradigm to simulate estrogen positive feedback. In the ARH, estradiol affected a wide spectrum of genes related to cell communication, metabolism, growth, transcription, and translation [20–22]. These studies examined the estradiol- transcriptome at a single time-point following systemic estradiol treatment and revealed a number of genes related to the regulation of reproduction: (e.g., *Pgr*, *Kiss1*, *Arc*, *Pomc*, *Pdyn*, and *Tac2)*.

The present study compared the temporal pattern of the transcriptome following estradiol treatment in both the AVPV region (AVPV+) and the ARH region (ARH+), cell groups responsible for positive and negative feedback, respectively. The estradiol coordination of activity in the ARH and AVPV is necessary for an appropriate GnRH response, as is gene expression in each region. To determine estradiol-induced transcriptomic responses, we examined the region-specific (AVPV+ and ARH+) and time-dependent responses. We compared estradiol regulation of gene expression between AVPV+ and ARH+ at specific time points after EB treatment, which is a novel approach to studying expression changes within the hypothalamus. Comparisons between time points allowed insights into how the temporal responses to estradiol on transcription differ between the AVPV+ and ARH+.

## Methods

### Ethics statement

This study was carried out in accordance with the principles and procedures of the National Institute of Health Guide for the Care and Use of Laboratory Animals. The protocol was approved by the Chancellor’s Animal Research Committee at the University of California, Los Angeles (Protocol Number: ARC # 2000–077).

### Animals

Ovariectomized (ovx) adult (~ 8 weeks of age) female Long-Evans rats (200–250 g) were purchased from Charles River (Wilmington, MA). Rats were shipped one-week post-ovariectomy. Upon arrival, rats were housed two per cage in a climate-controlled room, with a 12-hr light, 12-hr dark cycle (lights on at 0600). The hormone injections were started two weeks after the ovariectomies. Food and water were available ad libitum to the rats.

### Steroid priming

**Fig 1:**
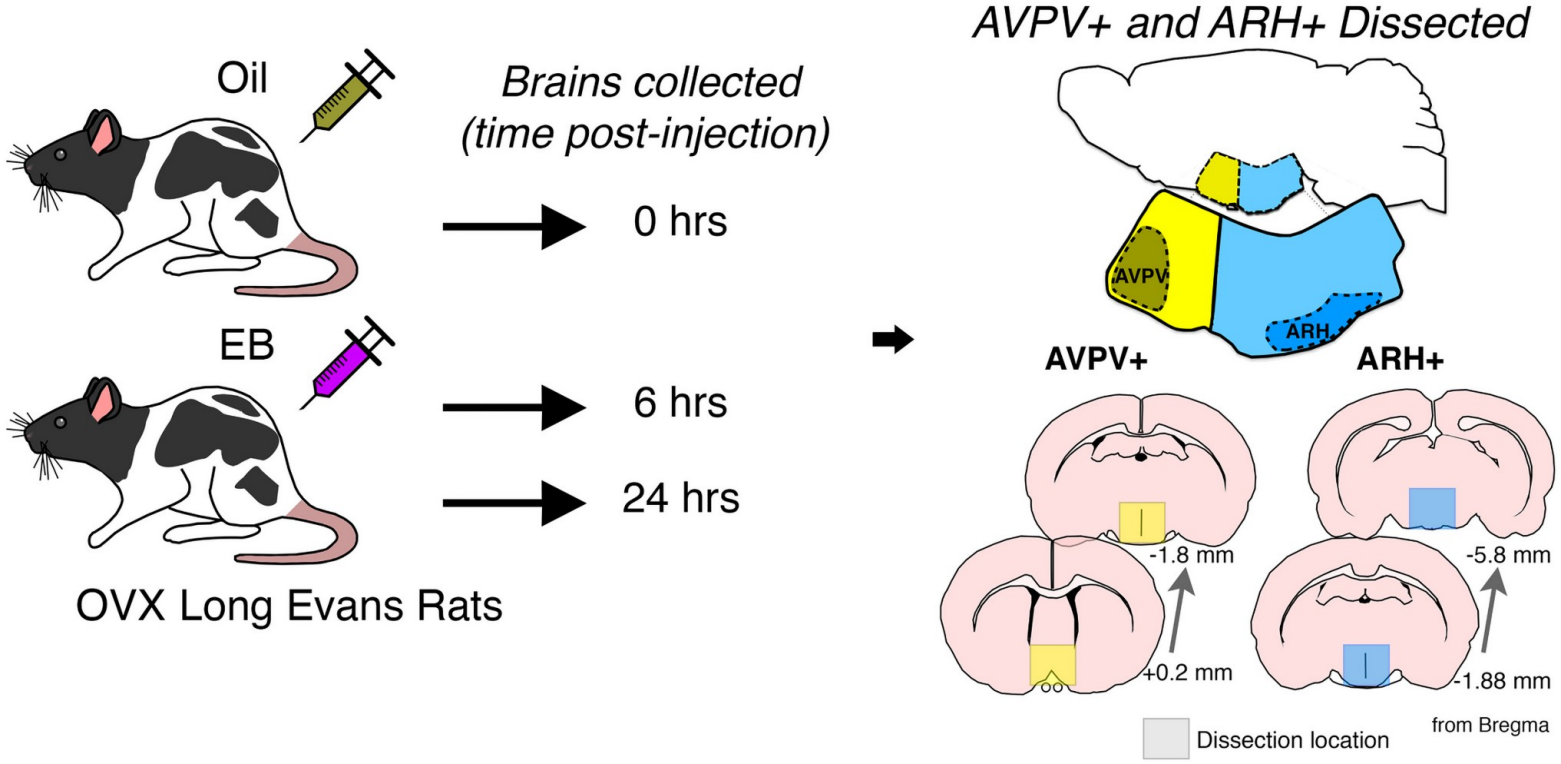
Experimental timeline and location of tissue dissections. Female rats received 5 μg EB in safflower oil every 4 days to mimic the natural estrous cycle for a total of 3 cycles. 6 or 24 hrs after the last EB injection, brains were collected. Baseline/oil-treated animals received just safflower oil for the 3 cycles and brains were collected immediately after the last oil injection. The AVPV+ and ARH+ were microdissected using the boundaries indicated in yellow and blue, respectively, and snap frozen for subsequent RNA isolation. The dissection locations are indicated in a sagittal view as well as representative schematics depicting coronal sections from Bregma, according to the Paxinos Rat Brain Atlas [24].

Estrogen injections began 2 weeks after ovariectomies. 17β-estradiol benzoate (EB) dissolved in safflower oil was injected (subcutaneously, s.c.) in a volume of 0.1 ml per rat. Females received 5 μg EB every 4 days between 0800 and 0900 for three cycles to mimic the natural estrous cycle of female rats as previously described [23]. Rats were anesthetized with isoflurane 6 hrs (n = 6) or 24 hrs (n = 6) after the third EB injection and brains were quickly isolated after decapitation. Baseline rats received oil for 3 cycles and sacrificed immediately following the last oil injection (n = 6). AVPV+ and ARH+ tissue was microdissected over ice using a matrix and razor blades (Fig 1) and then quick-frozen and stored at -80°C.

### RNA isolation

Total RNA from microdissected AVPV+ and ARH+ were isolated using TRIzol reagent (Life Technologies; Carlsbad, CA), according to a protocol recommended by the manufacturer. Briefly, tissue was homogenized in TRIzol^®^ at a ratio of 100 mg tissue/1 mL TRIzol^®^ (Thermo Fisher). Following the chloroform extraction of RNA and precipitation with 100% isopropanol, RNA pellet was washed with 75% ethanol/DEPC-treated water. Pellets were allowed to dry for 10 min at room temperature and were resuspended in 100 μL DEPC water. RNA samples were further purified using the E.Z.N.A. MicroElute RNA Clean Up Kit (Omega Bio-Tek; Norcross, GA) according to manufacturer’s instructions. Briefly, 250 μL QVL Lysis Buffer was added to each RNA sample and subsequently applied to a MicroElute LE RNA Mini Column. The diluent was separated from the RNA bound to the silica column by centrifugation at 10,000 X g for 15 seconds. After washing columns twice with RNA Wash Buffer II, RNA was eluted from the column with 15 μL of DEPC water. RNA concentrations and quality were reassessed using the NanoDrop^™^ 1000 spectrophotometer (Thermo Scientific, Waltham, MA). RNA concentration was >200 μg/mL, 260/280 (nucleic acid/protein) ratio was between 1.8 and 2.0 and 260/230 (nucleic acid/organic contaminants) ratio was between 2.0 and 2.2.

### RNA-seq

To test for RNA integrity, samples were initially analyzed on a denaturing agarose gel (1% agarose in 1X MOPS buffer (20 mm MOPS, 5 mM sodium acetate, 1 mM EDTA, 1 mM EGTA) plus 6.7% formaldehyde). Briefly, 0.5-3X volumes of formaldehyde loading dye (62.5% deionized formamide, 1.14 M formaldehyde, 200 μg/ml bromphenol blue, 1.25X MOPS buffer, 0.5% ethidium bromide) was added to 1 μg RNA and samples were heated at 65°C for 10 min. Samples were run on the gel at 5–6 V/cm^2^. Gels were visualized on a UV illuminator (Fluor ChemE imager; ProteinSimple, San Jose, CA). Intact total RNA samples with sharp 28S and 18S rRNA bands (28S rRNA band was approximately twice as intense as the 18S rRNA band) were further analyzed. Samples were removed when these criteria were not met. Final sample sizes were as follows: ARH baseline n = 6; ARH 6 hr n = 6; ARH 24 hr n = 5; AVPV baseline n = 4; AVPV 6 hr n = 4; AVPV 24 hr n = 4.

RNA integrity was also assessed using automated capillary electrophoresis on a Fragment Analyzer (Advanced Analytical Technologies, Inc., Ames, IA). Total RNA input of 500ng was used for library preparation using the Truseq Stranded mRNA Library Preparation Kit (Illumina; San Diego, CA, USA). Sequencing libraries were quantified by PCR using KAPA Library Quantification Kit for NGS (Kapa; Wilmington, MA, USA) and assessed for size distribution on a Fragment Analyzer. Sequencing libraries were pooled and sequenced on a HiSeq3000 (Illumina) using 150 cycle SBS kit with paired-end reads at 76 bp length [25, 26].

### Read mapping and quantification

Paired end reads were mapped to the *Rattus norvegicus* genome (Rnor6.0) using STAR v2.5.0a [27] with default settings. Read counts were quantified with HTSeq using union exon models [28]. We obtained 27–45 million uniquely mapped reads for each sample.

### Differential gene expression analysis

Differential gene expression analysis was performed using the DESeq2 R package [29]. Genes that were detected in at least two samples with at least 20 total read counts were used for this analysis. Expression values were normalized for library size, and differential expression analysis was performed using the DESeq function. In S1 Table, base mean is defined as the mean of normalized counts across all samples, normalizing for sequencing depth using the median of ratios method in DESeq2. Counts per million and standard deviation values for all genes used in differential gene expression analysis are provided in S1 Table. Genes with an FDR (adjusted p value) < 0.1; fold change > = |1.3|; p value < 0.01 were considered to be significantly differentially expressed. Volcano plots of genes were made in R, with -log_10_(adjusted p value) plotted on the y-axis and log_2_(fold change) plotted on the x-axis. Genes highly differentially expressed and/or genes of interest were labeled using Adobe Illustrator (HQ). Total numbers of significantly differentially expressed genes were plotted using GraphPad Prism 7 and Venny 2.1 was used to visualize the overlaps of significantly differentially expressed genes [30].

### Weighted gene coexpression network analysis (WGCNA)

The WGCNA R package [31] was used for building signed coexpression networks. Biweight midcorrelation was first used to calculate pair-wise correlations between genes. Next, pair-wise topological overlap was calculated with a power of 14 based on a fit to scale-free topology. Coexpression modules comprised of positively correlated genes with high TO were then identified using the cutreeDynamic function in the dynamicTreeCut R package [31], with the following parameters: method = "hybrid", deepSplit = 4 for ARH and 2 for AVPV+ and all samples from both regions, pamStage = F, minClusterSize = 50 for ARH and 100 for AVPV+ and all samples from both regions. The expression of each module was summarized by the module eigengene (ME, defined as the first principal component of all genes in a module). Modules whose eigengenes were highly correlated were further merged using the mergeCloseModules function in the WGCNA R package. Pearson correlations between MEs and experimental conditions (treated as binary numeric variables) were calculated. p values were FDR adjusted using Benjamini-Hochberg (BH) correction.

### Gene ontology analysis

Gene ontology analysis and functional classification were performed for genes in individual co-expression modules identified in WGCNA (module membership > = 0.5, p < 0.05) using the R package *goseq* [32], with gene length corrected and all genes expressed used as background. Significance was set at BH adjusted P-value < 0.1. REVIGO50 was used to cluster enriched GO-terms in modules significantly affected by estradiol. GO-terms were visualized by log10 p-value (Y-axes) and Resnik (normalized) semantic similarity of GO-terms (X-axes). Relevant GO-terms were selected in the scatterplots.

## Results

### Genes differentially expressed between the AVPV+ and ARH+ at specific post-EB time points

**Fig 2:**
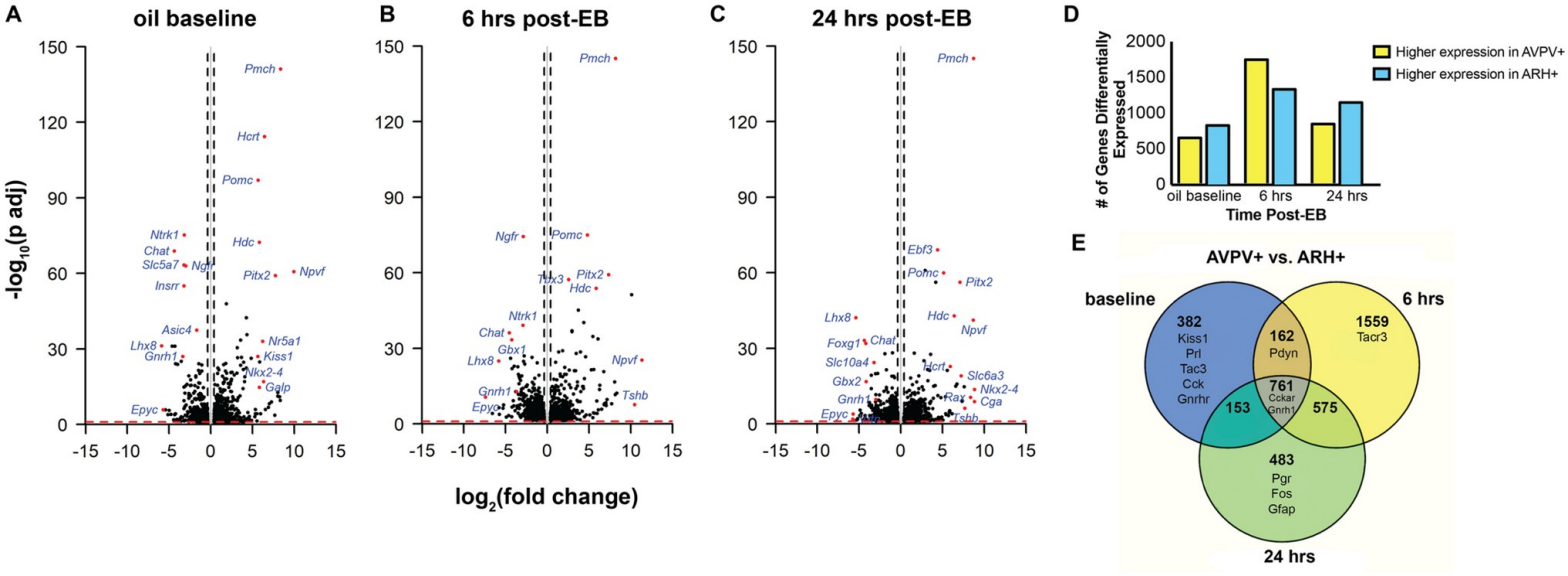
Differential gene expression analysis comparing gene expression levels in the AVPV+ compared with the ARH+ at oil baseline, 6 hrs, and 24 hrs post-EB. (A-C) Volcano Plots illustrating fold-change (log base 2) plotted against FDR-adjusted p value (- log base 10) of genes differentially expressed between brain regions at each time post-EB. Horizontal bar at y = 1 represents a significance level of FDR<0.1, p <0.01, and vertical lines indicate fold change > = |1.3| (dots below and between these lines did not reach our threshold of significance). (D) Bar graph depicting the numbers of genes with significant differential expression between the AVPV+ and ARH+ at different times post-EB treatment. (E) Venn diagram presents the overlap of 874 genes that are differentially expressed at each time point post-EB comparing expression in the AVPV+ to the expression in the ARH+.

To investigate the effects of estradiol on gene expression between the AVPV+ and ARH+, differential gene expression analysis based on the negative binomial distribution was performed. Expression levels of genes in the AVPV+ were compared to expression levels in the ARH+ at oil baseline, 6 hrs post-EB, and 24 hrs post-EB (Fig 2). In each estradiol condition, genes such as *Pomc* and *Npvf* were very strongly upregulated in the ARH+, whereas genes such as *Gnrh1* and *Chat* were very strongly upregulated in the AVPV+. The top 10 genes most differentially between the AVPV+ and ARH+ (i.e., highest positive and negative logarithmic fold change) are indicated in Table 1.

**Table 1:** Top DE genes between AVPV+ and ARH+ (positive FC = higher in ARH+, negative FC = higher in AVPV+).

baseline				6 hrs post-EB				24 hrs post-EB			
*log2(FC)*	*gene*	*base Mean*	*p value*	*log2(FC)*	*gene*	*base Mean*	*p value*	*log2(FC)*	*gene*	*base Mean*	*p value*
9.93	Npvf	998.87	1.20E-64	11.31	Npvf	726.91	7.50E-29	8.82	Cga	568.00	7.88E-12
8.35	Pmch	23575.41	4.51E-146	10.45	Tshb	148.55	2.73E-10	8.81	Nkx2-4	47.46	6.37E-17
8.30	Cga	1083.60	2.08E-12	10.09	Cga	1110.68	2.47E-55	8.72	Pmch	25429.85	1.90E-210
8.24	Rax	54.18	6.56E-14	8.30	Pitx1	33.53	3.59E-15	8.66	Npvf	822.11	3.04E-45
8.04	Slc6a3	419.00	1.09E-15	8.15	Pmch	20404.81	5.99E-151	8.34	Rax	56.51	1.20E-13
8.02	Foxa1	28.27	3.88E-14	8.14	Foxa1	29.83	1.91E-14	7.67	Tshb	21.49	7.91E-09
7.75	Pitx2	348.38	4.11E-63	8.06	Slc6a3	565.81	6.87E-20	7.58	Nr5a1	82.56	3.84E-18
7.66	Fshb	21.97	6.14E-06	7.71	Fshb	22.28	1.29E-10	7.34	Foxa1	17.05	5.15E-12
7.26	Slc15a5	16.77	8.52E-12	7.34	Pitx2	300.99	1.51E-63	7.23	Slc6a3	174.52	1.13E-22
7.00	Vgll2	13.90	5.36E-11	7.15	Nkx2-4	41.12	1.94E-12	7.07	Pitx2	233.22	2.45E-60
-5.90	Lhx8	460.08	6.57E-35	-7.40	Epyc	17.60	1.50E-13	-5.73	Vrtn	4.16	1.06E-03
-5.72	Epyc	3.87	1.86E-08	-6.56	SNORD52	7.06	2.41E-06	-5.71	Epyc	9.35	2.99E-06
-5.42	Opn5	6.37	2.42E-08	-5.81	Lhx8	455.00	1.56E-28	-5.37	Lhx8	518.41	3.11E-46
-5.14	Vrtn	2.63	3.18E-06	-5.78	Vrtn	8.12	5.74E-09	-5.32	Lpcat2b	3.14	2.56E-03
-4.97	Nr1h4	6.51	4.57E-08	-5.73	Arr3	3.93	4.85E-06	-5.18	Arr3	4.38	1.40E-03
-4.75	Umodl	4.05	2.03E-06	-5.32	Nr1h4	5.95	1.02E-08	-5.16	Hal	4.28	5.81E-04
-4.62	Foxg1	704.03	8.53E-35	-5.24	Opn5	10.63	3.84E-10	-4.90	Gsx2	7.89	1.56E-05
-4.45	AABR07064681.1	1.61	8.29E-04	-4.90	Lpcat2b	4.54	1.08E-04	-4.87	Ankrd63	63.62	5.34E-08
-4.38	Akr1c2	2.23	1.01E-03	-4.64	Hal	5.29	2.40E-05	-4.81	Umodl	5.15	3.34E-05
-4.37	Chat	604.68	4.74E-73	-4.53	Chat	552.12	6.13E-40	-4.72	Opn5	9.25	1.44E-06

Top genes differentially expressed between the AVPV+ and ARH+. A positive FC indicates a gene whose expression is higher in the ARH+, and a negative FC indicates a gene whose expression is higher in the AVPV+.

To better visualize the total numbers of differentially expressed genes between the AVPV+ and ARH+, genes that were significantly differentially expressed at each time post-EB were summarized in a bar graph (Fig 2D). At baseline (oil) 57% of genes were upregulated in the ARH+ compared with the 43% that were upregulated in the AVPV+ (826 genes higher in ARH+ vs. 632 genes higher in AVPV+). At 6 hrs post-EB, expression patterns changed. Differentially expressed genes increased dramatically with 56% of genes upregulated in the AVPV+ compared with 44% upregulated in the ARH+ (1726 genes higher in AVPV+ vs. 1330 genes higher in ARH+). Twenty-four hours post-EB treatment the percentages of differentially expressed genes between AVPV+ and ARH+ fell. In the AVPV+, 42% were upregulated and in the ARH+, 58% were upregulated (825 genes higher in AVPV+ vs. 1146 genes higher in ARH+).

Venn diagram analysis compared the overlap of genes whose expression was significantly different between the AVPV+ and the ARH+ at each of the three estradiol conditions (Fig 2E). Genes such as *Kiss1*, *Prl*, *Tac3* (encoding NKB, aka rodent *Tac2*, Ensembl:ENSRNOG00000004229), *Cck*, and *Gnrhr* were of the 382 genes that were different between the AVPV+ and ARH+ at baseline. Six hours post-EB genes such as *Tacr3* were differentially expressed between the AVPV+ and ARH+. *Pgr* was differentially expressed between the AVPV+ and ARH+ but only 24 hrs post-EB. Some genes, regardless of EB treatment, were differentially expressed between the AVPV+ and ARH+, including *Cckar*, *Gnrh1 and Cyp19a1*. Differences in *Gnrh1* provided an internal validation of our dissections–that they consistently captured these hypothalamic nodes.

### The transcriptomic response to EB is temporally different in the AVPV+ and ARH+

**Fig 3:**
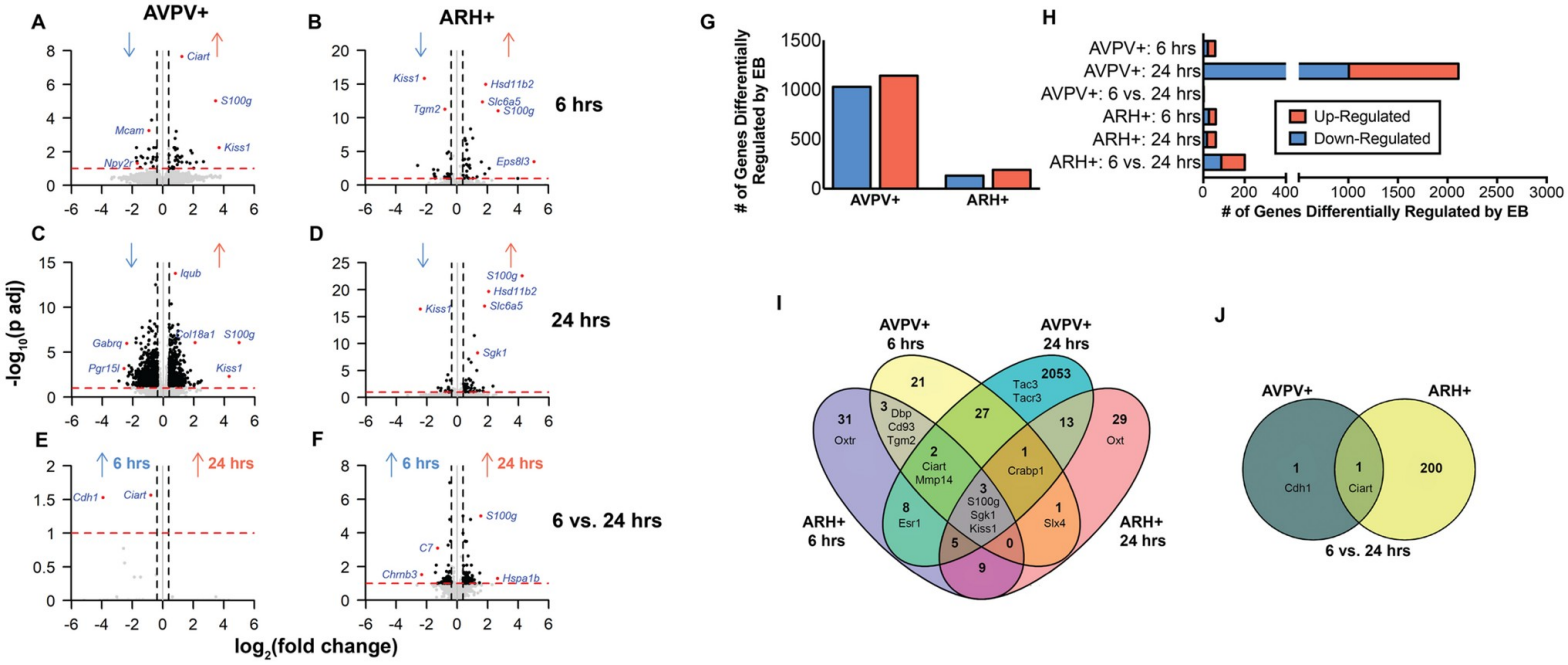
Differential gene expression analysis comparing the temporal responses to EB-induced transcription in the AVPV+ and ARH+, by comparing expression levels at baseline with 6 hrs post-EB and 24 hrs post-EB within each brain region. (A-F) Volcano Plots illustrating fold-change (log base 2) plotted against FDR-adjusted p value (- log base 10) of genes differentially expressed between EB treatment conditions. Note that in the baseline ARH vs. AVPV plot, -log10(adjusted p value) of *Pmch* was set to 145 so all points could be plotted within the 0–150 range. Horizontal bar at y = 1 represents a significance level of FDR<0.1, p <0.01, and vertical lines indicate fold change > |1.3| (grey dots did not reach our threshold of significance). (G) Overall (i.e., regardless of time post-EB), EB causes greater up-regulation of genes in the AVPV+ and the ARH+ (chi square df = 7.614, 1; z = 2.759, p <0.0058). EB causes higher differential expression of genes in the AVPV+ compared with the ARH+. (H) In the AVPV+ the highest number of genes differentially expressed by EB is observed 24 hours post-EB. In the ARH, the highest number of genes differentially expressed by EB occurs between 6 and 24 hours post-EB. (I) Venn diagram depicting the overlap of genes differentially expressed by estradiol relative to baseline 6 hrs post-EB, 24 hrs post-EB in both the AVPV+ and ARH+. (J) Venn diagram depicting the overlap of genes differentially expressed between 6 and 24 hrs post-EB in both the AVPV+ and ARH+.

To investigate the temporal effects of estradiol on gene expression in AVPV+ and ARH+, differential gene expression analysis based on the negative binomial distribution was performed. Pairwise comparisons within the AVPV+ and ARH+ were performed 6 hrs post-EB and 24 hrs post-EB (Fig 3). First, we investigated expression profiles from AVPV+ and ARH+ at these times (Fig 3A–3F). Estradiol strongly up regulated *Kiss1* in AVPV+ (Fig 3A and 3C) and down regulated *Kiss1* in ARH+ (Fig 3B and 3D). In distinction, *S100g* was very strongly up regulated by estradiol in both the AVPV+ and ARH+. The top 10 genes whose expression was most affected by estradiol (i.e., highest positive and negative logarithmic fold change) are indicated in Tables 2–4. Comparing differentially expressed genes between oil and 6 hrs post-EB, *Kiss1* was the most up- regulated DE gene in the AVPV+, while it was the 2^nd^ most down-regulated gene in the ARH+ (Table 2). The differential expression of *Kiss1* by observed at 6 hrs and remained changed after 24 hrs: the second most up-regulated gene in the AVPV+ and the most down-regulated gene in the ARH+ at 24 hrs (Table 3).

**Table 2:** Top DE genes baseline vs 6 hrs post- EB.

AVPV+				ARH+			
*log2(FC)*	*gene*	*base Mean*	*p value*	*log2(FC)*	*gene*	*base Mean*	*p value*
3.70	Kiss1	109.75	2.98E-06	5.06	Eps8l3	8.38	3.98E-07
3.47	S100g	24.07	1.09E-09	3.98	Crisp3	4.11	4.43E-04
2.66	Lrrc39	9.96	9.78E-05	2.70	S100g	63.94	2.75E-15
2.20	Epyc	27.40	1.16E-04	2.11	Rn50_X_0645.1	7.79	6.68E-06
2.03	Mmp3	13.29	3.48E-04	1.88	Hsd11b2	40.17	1.30E-19
1.91	Gpx2	20.53	1.26E-05	1.83	Pnpla1	112.53	4.89E-05
1.84	Col18a1	1461.21	8.57E-06	1.67	Slc6a5	73.43	8.00E-17
1.77	Ms4a2	16.75	1.40E-04	1.60	Oxtr	24.74	4.60E-07
1.56	AABR07030914.1	123.96	1.17E-04	1.32	Akr1b7	16.35	2.15E-04
1.26	Ifit3	55.97	1.20E-04	1.06	Fmo2	22.86	3.59E-04
-1.74	Nr4a3	108.47	4.03E-05	-2.57	AABR07014424.1	6.89	1.85E-06
-1.73	Mmp14	192.76	3.54E-06	-2.13	Kiss1	464.28	8.72E-21
-1.70	Cd93	199.75	9.11E-05	-1.54	Hbb	766.16	9.78E-06
-1.67	Npy2r	123.45	1.47E-04	-1.50	LOC103694857	459.30	6.03E-05
-1.48	LOC100909913	15.16	2.96E-04	-1.48	Hba-a2	884.67	5.35E-05
-1.42	Pcdh12	39.70	2.72E-05	-1.46	LOC103694855	212.85	1.40E-04
-1.32	Primpol	32.15	4.26E-05	-1.43	Alas2	15.87	4.22E-04
-1.14	Tgm2	338.62	1.57E-04	-1.41	Hba-a2	332.22	2.01E-04
-1.01	Adamts4	1423.37	4.98E-05	-1.26	Col6a5	56.60	1.18E-05
-0.92	Adamts1	234.43	5.80E-06	-0.81	Mmp14	214.79	1.98E-04

Differentially expressed genes with the highest and lowest fold change from oil to 6 hrs post-EB in the AVPV+ and ARH+. A positive FC indicates a gene whose expression is higher at 6 hrs post-EB, and a negative FC indicates a gene whose expression is lower at 6 hrs post-EB.

**Table 3:** Top DE genes baseline vs 24 hrs post- EB.

AVPV+				ARH+			
*log2(FC)*	*gene*	*base Mean*	*p value*	*log2(FC)*	*gene*	*base Mean*	*p value*
4.97	S100g	60.87	2.37E-09	4.24	S100g	157.11	2.76E-23
4.31	Kiss1	156.15	2.51E-04	2.33	Ms4a8	7.42	8.69E-03
2.49	Wdr64	11.18	1.30E-03	2.04	Hsd11b2	41.59	2.12E-20
2.40	Slc22a7	29.61	2.30E-03	1.78	Slc6a5	74.09	1.08E-17
2.28	AABR07012039.1	11.87	1.91E-03	1.70	Wdr64	13.40	1.73E-02
2.26	Mmp3	14.34	3.46E-03	1.68	Ranbp3l	151.77	6.25E-02
2.07	Col18a1	1578.84	2.52E-09	1.45	Fam159a	20.50	5.31E-02
2.02	Tcp11x2	19.73	4.94E-03	1.32	Sgk1	1056.23	5.34E-09
2.00	Ihh	11.23	8.79E-03	1.29	LOC103690541	26.44	5.75E-02
1.79	LOC108352861	18.03	2.81E-05	1.26	Nxf3	55.24	1.01E-05
-2.91	Slc26a7	18.81	1.45E-03	-2.44	Kiss1	478.30	9.40E-21
-2.57	Pgr15l	66.69	1.37E-05	-1.26	AABR07067600.1	15.02	3.41E-05
-2.39	Gabrq	116.85	3.67E-09	-1.11	RGD1563601	315.83	2.07E-04
-2.39	Angptl2	37.08	3.69E-03	-1.07	Col6a5	61.24	4.18E-05
-2.32	Cnn1	14.12	5.96E-03	-0.78	Ubxn10	95.02	3.32E-04
-2.31	C7	100.05	2.27E-04	-0.78	Hhat	31.65	1.94E-04
-2.25	Met	14.46	5.10E-06	-0.71	Slc7a11	948.30	6.63E-07
-2.23	Dgkk	42.69	5.54E-05	-0.63	Igfbp5	3769.06	3.79E-08
-2.22	Sstr5	17.17	1.05E-04	-0.61	Fgr	58.56	7.36E-05
-2.10	Gpr17	224.86	4.01E-05	-0.60	St6galnac5	199.51	2.44E-05

Differentially expressed genes with the highest and lowest fold change from oil to 24 hrs post-EB in the AVPV+ and ARH+. A positive FC indicates a gene whose expression is higher at 24 hrs post-EB, and a negative FC indicates a gene whose expression is lower at 24 hrs post-EB.

**Table 4:** Top DE genes 6 vs 24 hrs post-EB.

AVPV+				ARH+			
*log2(FC)*	*gene*	*base Mean*	*p value*	*log2(FC)*	*gene*	*base Mean*	*p value*
	none			2.65	Hspa1b	57.34	2.40E-04
				1.55	S100g	205.28	2.01E-09
				1.48	Tcp11x2	21.32	6.39E-05
				1.46	Y_RNA	52.50	1.40E-03
				1.15	Ube2c	18.67	1.95E-03
				1.13	SNORD14	35.77	3.62E-04
				1.09	AC130970.1	115.03	4.02E-04
				1.01	Adgrg3	22.81	1.26E-04
				0.99	AC135409.1	95.45	1.13E-04
				0.97	Hspa8	96.71	7.81E-04
-0.7955	Ciart	31.58	1.43E-06	-2.33	Chrnb3	22.20	9.53E-05
-3.916	Cdh1	332.72	3.13E-06	-1.42	LOC103693608	23.66	5.72E-05
				-1.37	Gpr17	177.04	8.63E-06
				-1.28	C7	81.24	6.71E-07
				-1.28	AABR07062799.2	52.16	2.03E-03
				-1.21	AABR07001068.1	53.75	1.46E-03
				-1.07	AABR07006310.1	353.45	1.27E-03
				-1.05	Pcdhga11	40.76	1.11E-03
				-1.04	AABR07040840.1	19.94	9.45E-04
				-1.00	RGD1563601	295.10	8.42E-04

Differentially expressed genes with the highest and lowest fold change from 6 to 24 hrs post-EB in the AVPV+ and ARH+. A positive FC indicates a gene whose expression is higher at 24 hrs post-EB compared with 6 hrs, and a negative FC indicates a gene whose expression is higher at 6 hrs post-EB compared with 24 hrs post-EB.

To compare the up and down regulation of genes by EB relative to baseline, the total numbers of differentially expressed genes were plotted (Fig 3G). In the AVPV+ and ARH+, more genes were up-regulated by EB (chi square df = 4.82, 1; z = 2.195, *p* = 0.0281; Fig 3G). In the AVPV+, 1142 genes (53%) and in the ARH+, 191 genes (59%) were up-regulated by EB. When less stringent criteria were used to determine whether genes were differentially expressed (e.g., *p* <0.05), similar numbers of genes are differentially regulated by EB (7823 in AVPV+ vs. 6595 in ARH+). The timing of EB-induced transcription in these two regions was different (Fig 3H). In the AVPV+ at 6 hrs post-EB 58 genes were differentially expressed (62% up, 38% down). Then, at 24 hrs post-EB there was a wave of transcription in the AVPV+ with 2111 genes differentially expressed (53% up and 47% down). The smallest number of transcriptional changes occurred in the AVPV+ between 6 and 24 hrs post-EB (2 differentially expressed genes, 0% up and 100% down-regulated). In stark contrast, the most transcriptional changes were observed in the ARH+ between 6 and 24 hrs post-EB (201 DE genes, 57% up and 43% down regulated). The same number of genes were differentially expressed in the ARH+ at baseline or 6 hrs post-EB (61 differentially expressed genes, 56% up and 44% down regulated) and baseline and 24 hrs post-EB (61 differentially expressed genes, 70% up and 30% down regulated).

Venn diagram analyses compared genes whose expression was significantly different at each time point compared to baseline (Fig 3I) and between 6 and 24 hrs post-EB (Fig 3J) in the AVPV+ and ARH+. For example, *Dbp*, *Cd93*, and *Tgm2* were differentially expressed after 6 hrs of estradiol treatment in both the AVPV+ and ARH+ relative to baseline. Only 3 genes, *S100g*, *Sgk1*, and *Kiss1*, were differentially expressed after EB relative to baseline at both 6 and 24 hrs in AVPV+ and ARH+ (Fig 3I). When comparing the expression of genes between 6 and 24 hrs post-EB, *Ciart* was differentially regulated in both the AVPV+ and ARH+ (Fig 3J).

For complete lists of overlapping genes, see S2–S4 Tables, and for details regarding each differential gene expression analysis, see S5–S13 Tables. Note that the region/time listed first in the file names for S5–S13 Tables is the denominator for each comparison.

### Weighted gene co-expression network analysis (WGCNA) revealed networks of genes modulated by EB in the AVPV+ and ARH+

**Fig 4:**
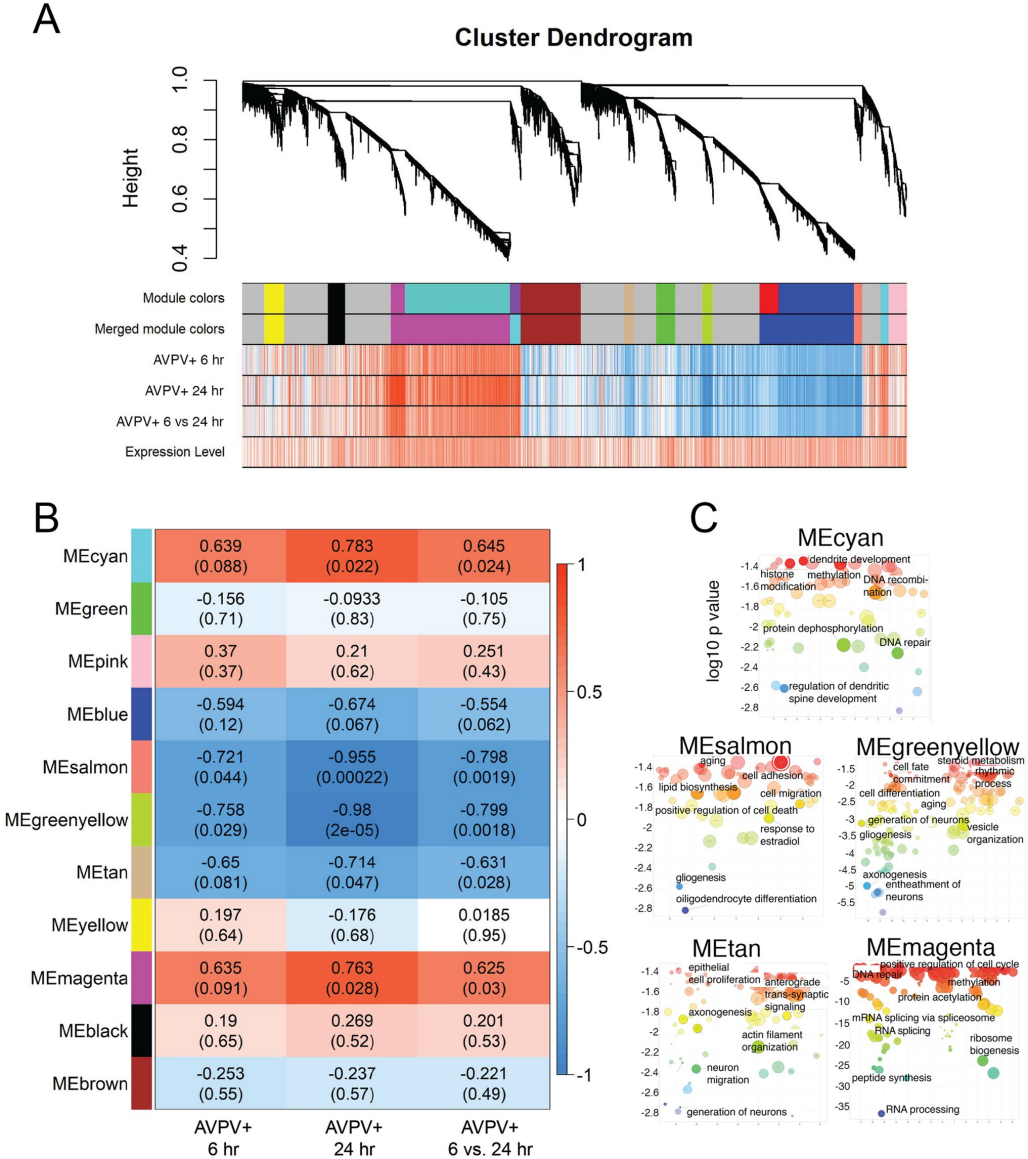
Gene network analysis: Clusters of genes modulated by EB in the AVPV+. (A) Cluster dendrogram demonstrating the hierarchical clustering of genes in the AVPV+ and heatmaps depicting the relative up- (red) and down-regulation (blue) of these genes by estradiol. (B) Heatmap showing Pearson correlation between gene coexpression modules defined by WGCNA in the AVPV+ and experimental conditions (6 hrs post-EB (compared to oil baseline), 24 hrs post-EB (compared to oil baseline) and 6 vs. 24 hrs post-EB). P values are indicated in parentheses. (C)GO-terms were visualized by log10 p-value (Y-axes) and Resnik (normalized) semantic similarity of GO-terms (X-axes).

**Fig 5:**
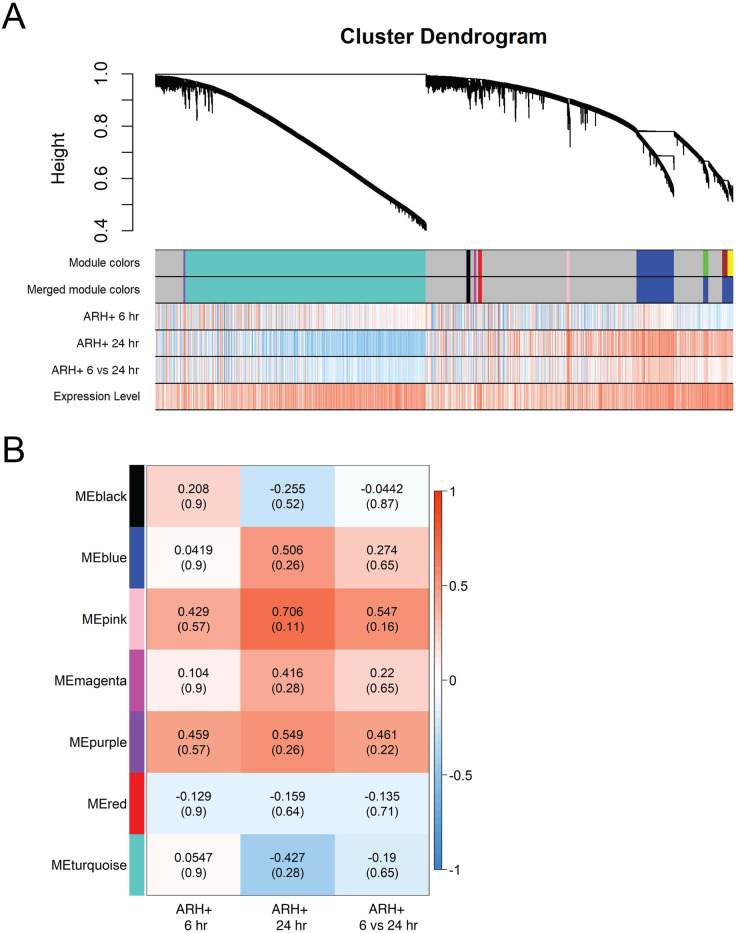
Gene network analysis: Clusters of genes modulated by EB in the ARH+. (A) Cluster dendrogram demonstrating the hierarchical clustering of genes in the ARH+ and heatmaps depicting the relative up- (red) and down-regulation (blue) of these genes by estradiol. (B) Heatmap showing Pearson correlation between gene coexpression modules defined by WGCNA in the ARH+ and experimental conditions (6 hrs post-EB (compared to oil baseline), 24 hrs post-EB (compared to oil baseline) and 6 vs. 24 hrs post-EB). P values are indicated in parentheses.

**Fig 6:**
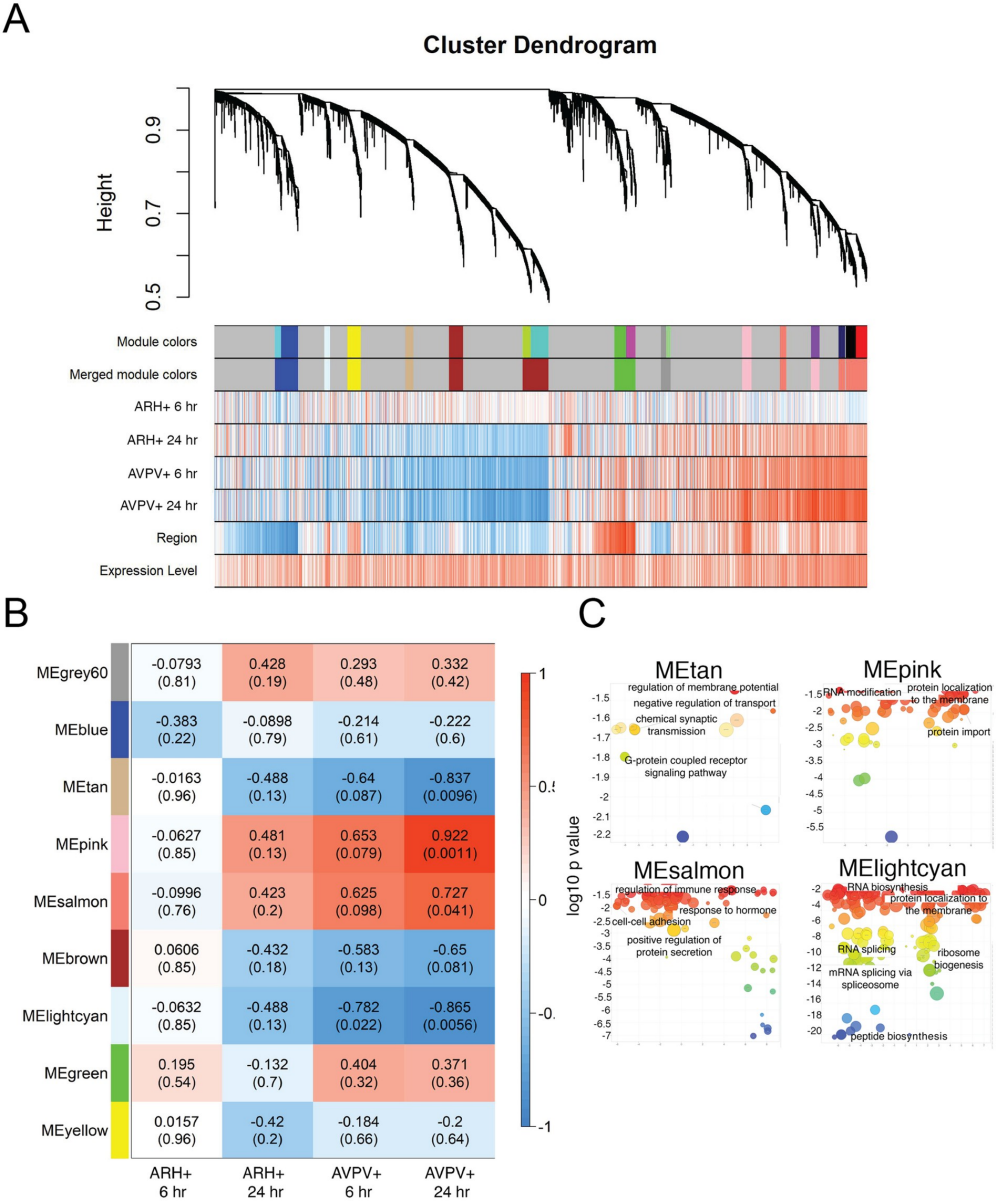
Gene network analysis: Clusters of genes modulated by estradiol in the combined AVPV+ and ARH+. (A) Cluster dendrogram demonstrating the hierarchical clustering of genes in the combined AVPV+ and ARH+ and heatmaps depicting the relative up- (red) and down-regulation (blue) of these modules by estradiol at 6 and 24 hrs post-EB, compared to oil baseline. (B) Heatmap showing Pearson correlation between gene coexpression modules defined by WGCNA in the combined AVPV+ and ARH+ dataset and experimental conditions (6 hrs post-EB (compared to oil baseline), 24 hrs post-EB (compared to oil baseline) and 6 vs. 24 hrs post-EB). P values are indicated in parentheses. (C) GO-terms were visualized by log10 p-value (Y-axes) and Resnik (normalized) semantic similarity of GO-terms (X-axes).

Weighted gene co-expression network analysis (WGCNA) was used to identify clusters (aka modules) of highly correlated genes modulated by EB from baseline to 6 hrs post-EB, from baseline to 24 hrs post-EB, and from 6 to 24 hrs-post EB within the AVPV+ (Fig 4), ARH+ (Fig 5), or the AVPV+ combined with the ARH+ (Fig 6). Gene networks were clustered into color-coded modules, whose response to EB was similar. AVPV+ modules in all comparisons had approximately equal up- or down-regulation (heatmaps Fig 4A and 4B) and had similar trends within modules. In the AVPV+, 11 modules were determined, with 5 modules responding significantly to EB (Fig 4B). REVIGO50 was used to cluster enriched GO-terms in modules significantly affected by EB (Figs 4C and 6C). Modules significantly affected by EB in the AVPV+ (Fig 4C), and AVPV+ and ARH+ together (Fig 6C), were assigned a function based on gene ontology. In the AVPV+, modules associated with dendrite spine development, RNA splicing and peptide synthesis, and epigenetic effects such as histone modification and methylation were significantly up-regulated by EB at 6 to 24 hrs post-EB and at 24 hrs. Simultaneously, modules associated with cell death, cell migration, and neuro- and gliogenesis were downregulated (Fig 4B and 4C).

ARH+ gene clusters had different temporal patterns of EB-induced up- and down-regulation. The heatmap patterns of up- and down-regulation by EB (Fig 5A) were vastly different when comparing ARH+ 6 hrs and ARH+ 24 hrs. Among the 7 modules in the ARH+, no modules were significantly up- or down-regulated by EB (Fig 5B).

Network analysis of combined gene expression in AVPV+ and ARH+ revealed 9 modules but only 4 modules were significantly affected by EB (Fig 6) and the effect was mostly driven by significant effects in the AVPV+. The tan, pink, salmon and light cyan modules showed significant up or down regulation by EB in the AVPV+, and not in the ARH+. The enriched GO-terms in each module varied when the AVPV+ was analyzed by itself (Fig 4C) compared to the combined ARH+ and AVPV+ (Fig 6C). For example, the magenta module in the AVPV+ analysis, which was up-regulated at 6 and 24 hrs post-EB contained enriched GO terms that included RNA splicing, peptide synthesis, and ribosome biogenesis, while the light cyan module in the combined analysis contains the same GO terms but is down-regulated at 6 and 24 hrs post-EB. For complete lists of genes within each module and for complete lists of GO-terms, see S14–S42 Tables.

## Discussion

Interestingly, but not unexpectedly, the patterns of gene expression in the AVPV+ and ARH+ were different in the baseline, oil-treated condition. This is most likely due to the different functions of these regions of the hypothalamus that may or may not be affected by estradiol. For example, the ARH is a complex nucleus implicated in the control of food intake, metabolism, cardiovascular regulation, and fertility, while the AVPV is mostly involved in the regulation of ovulation. Thus, the present results provide information about estradiol-induced transcriptional changes in different areas of the hypothalamus that contribute to homeostatic maintenance of gene networks that provide clues about their induction throughout the estrous cycle.

The AVPV and ARH are also critically important for the hypothalamic control of GnRH release, which estradiol regulates. Within the rodent ARH, estradiol exerts a control of GnRH pulsatility and inhibition of release (negative feedback). The AVPV appears less complicated and is concerned with the surge release of GnRH (positive feedback). As hypothesized, these regions have different transcriptomic responses to estradiol treatment. Estradiol induced both different patterns of up- or down-regulation of genes, but also which gene networks were affected. Because estrogen feedback has a specific temporal pattern, it was important that the temporal patterns of gene responses to estradiol were also distinct in each region. Much of the previous work on estradiol action in the AVPV that is associated with positive feedback suggested that there is a lag between EB treatment and the release of GnRH and the subsequent LH surge. The present results provide information about estradiol-induced transcriptional changes in different areas of the hypothalamus that contribute to homeostatic maintenance of gene networks throughout the estrous cycle.

High-throughput studies investigating the transcriptomic responses to estradiol in the AVPV+ or ARH+ have focused on the perinatal period [33, 34], exposure to endocrine disrupting chemicals during prepuberty [35], or on the ARH exclusively [22]. The current study is the first high-throughput analysis of the transcriptomic responses to estradiol in the adult female rat AVPV and ARH regions. Moreover, it is becoming clear that estradiol has more rapid actions than those that appear at 48 hours, the time point initially chosen because it correlated with estradiol induction of behavior (e.g., lordosis reflex) or physiological response (e.g., the LH surge). The goal of the present studies was to examine underlying changes in expression both at a regional level and at earlier time points before the final response. The data revealed that the hypothalamus responds very differently based on the region and time point after estradiol treatment suggesting a complex interplay of gene expression in the AVPV+ and ARH+ that regulates female reproductive patterns. Although we restricted our dissections to blocks containing the AVPV and ARH, we are cognizant that these dissections included surrounding tissue and influenced the response we observed. For example, *Pmch* is not expressed exclusively in the ARH but is also expressed in the lateral hypothalamus. Importantly, these studies confirmed several genes known to be regulated by estradiol (e.g., *Kiss1*, *Pgr*, *Prl*, *Esr1*) to indicate that our methods had sufficient power to have an accurate picture of estradiol-sensitive gene expression.

By comparing within animals (i.e., comparing the expression of genes between the AVPV+ and ARH+) and between animals (i.e., comparing the expression of genes after estradiol treatment), a more complete picture estradiol regulation of transcription in the hypothalamus emerged. For example, at baseline, *Kiss1* gene expression is 50-fold higher in the ARH+ compared with the AVPV+. It is well established that *Kiss1* expression in OVX oil-treated rats is higher in the ARH compared with the AVPV, and estradiol treatment decreases *Kiss1* expression in the ARH, but increases expression in the AVPV [8]. However, at 6 and 24 hrs post-EB, *Kiss1* is no longer differentially expressed between the two regions, but is differentially expressed within each region compared to baseline. That is, there are baseline differences in *Kiss1* expression, but after estradiol treatment *Kiss1* decreased in the ARH+ and increased in the AVPV+, such that the differences between regions disappeared. The observed patterns of up and down regulation of *Kiss1* by estradiol in the present study validated our approach.

Of the genes whose expression is most strongly affected by estradiol is *S100g*. S100 calcium-binding protein G, *S100g*, is a gene that encodes calbindin D9k, a cytosolic calcium-binding protein. There is a paucity of information about the role of *S100g* in the brain, although its protein product is expressed throughout the hypothalamus, brain stem, and cerebellum, and is colocalized with mature, GABAergic, dopaminergic, and oxytocinergic neurons in the prepubertal rat [36]. Calbindin D9k is expressed in female reproductive tissues and the pituitary gland where its levels fluctuate with estradiol levels and throughout the estrous cycle [37, 38]. The expression of *S100g* in rats depends on the activation of nuclear estrogen receptor-α binding to the estrogen response element within the promotor region of *S100g* [39, 40]. In the present study, the expression of *S100g* did not differ between the AVPV+ and ARH+, but its expression was highly sensitive to estradiol; *S100g* was the second and third gene most upregulated by estradiol at 6 hrs post-EB in the AVPV+ and ARH+, respectively. Six hours after estradiol treatment the *S100g* expression increased 6.5-fold in the ARH+ and 11-fold in the AVPV+, and at 24 hours after estradiol treatment, its expression increased 18-fold in the ARH+ and 31-fold in the AVPV+. At 24 hrs post-EB, *S100g* was the most upregulated gene in each region (Table 3). Based on a similar response in the AVPV+ and the ARH+, we surmise that it is unlikely that this gene is involved in the switch between negative and positive feedback. The estradiol-induced transcription of genes such as *S100g* could identify yet unknown candidates that play significant roles in the homeostatic functions of the hypothalamus.

While estradiol-regulated *Kiss1* gene expression followed patterns previously observed [8], expression of *Tac3* (encoding NKB) and *Tacr3* (encoding NKB receptor) in this study did not follow expected patterns. *Tac3* and *Tacr3* did not emerge as genes differentially expressed by estradiol in the ARH+, where a majority of kisspeptin neurons also express NKB [41]. Even though the ARH kisspeptin population co-expresses *Tac3*, in situ hybridization studies in OVX ewes failed to detect any estradiol-induced changes in *Tac3* mRNA expression in the ARH [42], but see [43]. Given the established role of *Tac3* in the ARH, we were surprised that 24 hrs after estradiol treatment, *Tac3* and *Tacr3* were differentially expressed in the AVPV+, such that *Tac3* expression doubles and *Tacr3* decreases by 60%, relative to oil baseline. To our knowledge, there is no known role of *Tac3* and *Tacr3* in the AVPV or close surrounding areas. Navarro et al. found that ~10% of AVPV Kiss1 neurons and ~11% of GnRH neurons express *Tac3* mRNA [44]. Recent evidence suggests that tachykinin signaling may be involved in the onset of puberty (reviewed in [45]).

In general, estradiol stimulated transcription of more genes in the AVPV+ compared with the ARH+. Estradiol rapidly altered transcription in the AVPV+ and ARH+. Within 6 hrs of EB treatment, we observe differential expression of hundreds of genes, and the majority of these genes were up-regulated. Interestingly, in the AVPV+, but not the ARH+, a larger second wave of gene transcription occurs 24 hours after EB treatment (i.e. thousands of differentially expressed genes 24 hrs post-EB compared with oil-treatment). However, when the gene expression in the AVPV+ at 6 hrs post-EB was compared to expression patterns at 24 hrs post-EB, only two genes met the criterion to be classified as significantly differentially expressed, and could be due to the variability of expression at 6 hrs. It is likely that if we had chosen different timepoints for our analysis the results would have been clearer. That said, the second wave of transcriptional changes may indicate networks of genes that underlie the switch to positive feedback. Cluster analysis revealed that at 24 hours after EB treatment modules involved in peptide synthesis and dendritic spine development are upregulated in the AVPV+, while modules involved in cell death and axonogenesis are down regulated. These networks of genes could regulate the dynamic relationship between glial cells and kisspeptin and GnRH neurons that are involved in the switch from negative to positive feedback [46].

These data contribute to a better understanding of how estradiol differentially affects transcription in hypothalamic loci that control different aspects of estrogen feedback, 6 and 24 hrs after estradiol treatment. While only hundreds of genes in the ARH+ are differentially expressed 6 and 24 hrs after estradiol treatment, thousands of genes in the AVPV+ are differentially expressed 24 hrs after estradiol treatment indicating the highly complex action of estradiol in the AVPV. Importantly, the up-regulation of gene transcription in one region with the coincident down-regulation of gene transcription in the other can mask the effects of estradiol if the hypothalamus is considered as one functional unit. Future studies will further elucidate how estradiol affects transcription involved in the switch between negative and positive feedback using single-cell sequencing on more specific dissections of the AVPV and ARH.

## Supporting information

S1 Table Counts per million. (CSV)

S2 Table AVPV ARH Venn. (XLSX)

S3 Table AVPV ARH oil 6 oil 24 Venn. (XLSX)

S4 Table AVPV ARH 6 24 Venn. (XLSX)

S5 Table DESeq2 results AVPV oil-ARH oil. (XLSX)

S6 Table DESeq2 results AVPV 6hr EB-ARH 6hr EB. (XLSX)

S7 Table DESeq2 results AVPV 24hr EB-ARH 24hr EB. (XLSX)

S8 Table DESeq2 results AVPV oil-AVPV 6hr EB. (XLSX)

S9 Table DESeq2 results ARH oil-ARH 6hr EB. (XLSX)

S10 Table DESeq2 results AVPV oil-AVPV 24hr EB. (XLSX)

S11 Table DESeq2 results ARH oil-ARH 24hr EB. (XLSX)

S12 Table DESeq2 results AVPV 6hr EB-AVPV 24hr EB. (XLSX)

S13 Table DESeq2 results ARH 6hr EB-ARH 24hr EB. (XLSX)

S14 Table GO module black AVPV. (CSV)

S15 Table GO module blue AVPV. (CSV)

S16 Table GO module brown AVPV. (CSV)

S17 Table GO module cyan AVPV. (CSV)

S18 Table GO module green AVPV. (CSV)

S19 Table GO module greenyellow AVPV. (CSV)

S20 Table GO module grey AVPV. (CSV)

S21 Table GO module magenta AVPV. (CSV)

S22 Table GO module pink AVPV. (CSV)

S23 Table GO module salmon AVPV. (CSV)

S24 Table GO module tan AVPV. (CSV)

S25 Table GO module yellow AVPV. (CSV)

S26 Table GO module black ARH. (CSV)

S27 Table GO module blue ARH. (CSV)

S28 Table GO module magenta ARH. (CSV)

S29 Table GO module pink ARH. (CSV)

S30 Table GO module purple ARH. (CSV)

S31 Table GO module red ARH. (CSV)

S32 Table GO module turquoise ARH. (CSV)

S33 Table GO module blue all. (CSV)

S34 Table GO module brown all. (CSV)

S35 Table GO module green all. (CSV)

S36 Table GO module grey all. (CSV)

S37 Table GO module grey60 all. (CSV)

S38 Table GO module lightcyan all. (CSV)

S39 Table GO module pink all. (CSV)

S40 Table GO module salmon all. (CSV)

S41 Table GO module tan all. (CSV)

S42 Table GO module yellow all. (CSV)
